# 

**Published:** 1894-12-01

**Authors:** 


**Notices of Births, Deaths, and Marriages** are inserted in this column at a charge of 2s. 6d. for an announcement not exceeding four lines.

**Notice to Advertisers and Subscribers.**

All Advertisements, Orders for Copies of The Hospital, and Business Communications should be addressed to **The Publisher, NOT TO THE EDITOR,** The Hospital, 428, Strand, W.C.

The Cost of Subscription, post free, is as follows:—Per week, 2½d.; per quarter, 2s. 8d.; per half-year, 5s. 3d.; per annum, 10s. 6d. Foreign:—Per Annum, 12s. 8d.; payable, in all cases, in advance.

### NOTICE TO CORRESPONDENTS.

All MS., Letters, Books for Review, and other matters intended for the Editor should be addressed The Editor, The Lodge, Porchester Square, London, W.

The Editor cannot undertake to return rejected MS., even when accompanied by stamped directed envelope.

**"Burdett's Hospital Annual," 1894.**

Fifth year of publication. 900 pp. Boards, gilt lettering. A most complete guide to British, Colonial, Indian, and American Hospitals, Dispensaries, Nursing and Convalescent Institutions, and Asylums. Price 5s. Published by The Scientific Press (Limited), 428, Strand, W.C.

**NOTICE.—The Index for Vol. XVI. (ending September 24th, 1894) is on sale at the Office of this Journal, Price 2½d., post free.**

### Scraps and Gleanings.

The Bournemouth Hospital Sunday Fund collections amounted to £627 this year.

The Kent County Ophthalmic Hospital, which is entirely free, treated 2,832 patients during the past year.

The scheme to extend the accommodation at the Dundee Eye Infirmary includes the provision of an ophthalmic ward.

The Leicester Infirmary has received £895 through the Hospital Sunday and £2,619 through the Hospital Saturday Fund collectors respectively.

The Leicester Infirmary has become the recipient of £895 through the local Hospital Sunday collection, and of £2,619 from the Hospital Saturday Fund.

It is stated that one fact demonstrated at the Buda-Pesth Congress was that the number of stammerers of the male sex largely exceeds those of the female.

In spite of depression of trade the working men of Coventry have handed over £1,000 to their hospital, the result of this year's Hospital Saturday collection.

No less a sum than £23,600 has been raised in Bradford by the collections in mills and workshops, in the course of the last twenty years, towards local charities.

Finances at the Axminster Cottage Hospital are not flourishing, and Miss Conybeare, the foundress, is somewhat despondent at the want of interest shown in the neighbourhood.

The balance-sheet of the house-to-house collection in favour of the Grantham Hospital shows receipts amounting to £172, an amount slightly in excess of the subscription of the previous year.

A novel mode of repressing drunkenness is being tried in St. Petersburg. The names of all persons found intoxicated by the police are placarded in public places and published in the *Official Gazette*.

At the annual meeting of the Glasgow Eye Infirmary it was announced that Mr. Anderson Rodger has generously contributed the sum of £383, being the sum expended above the estimated cost for the new buildings.

A scheme is being considered which will enable the Devizes Cottage Hospital to secure free relief to patients who earn less than thirty shillings weekly or for members of the family of the bread-winner to the same extent.

It is intended to provide extra accommodation for the nurses and servants at the Royal Isle of Wight Infirmary. The addition of a lift is also contemplated, but these improvements must await the collection of the necessary funds.

A new operating room is to be built at the Northampton General Infirmary, Messrs. Young and Hall being the architects. Mrs. Phipps, of Collingtree, has already promised £500 towards the scheme, which is estimated to cost £2,000.

The Convalescent Home at Bosgrove, Berkshire, is to be continued as such by Mrs. Wilder, of Sulham House. Ten beds will be available for the reception of patients. Previously the home was used for patients from St. Mary's Hospital.

The scheme to extend the Sheffield Public Hospital has received pecuniary support to the extent of £25,000. This is half the sum required, and the collectors are well satisfied at the progress made. A house to house collection by ladies is to follow.

At a meeting in aid of the extension of the Devon and Exeter Hospital, it was announced that Mr. Dawson was willing that his donation of £3,000 should be applied to the building fund, and that £400 were promised towards the same object.

A special plea is being made for increased aid to the Torbay Hospital. This admirable institution having been increased and enlarged entails heavier annual expenditure. Over four hundred persons now annually pass through this particular hospital.

Lord Russell of Killowen (Lord Chief Justice of England) has consented to preside at the festival dinner in aid of the funds of the City of London Hospital for Diseases of the Chest, which is arranged to take place at the Metropole Hotel, April 30th, 1894.

The annual meeting of the Dudley Dispensary has just been held, the report of which states that the adverse balance brought forward from the previous year, together with some extraordinary expenses during the present year, prevent the committee from presenting as favourable a report as usual.

The last quarterly meeting of the Newcastle Infirmary showed a large increase of surgical patients, necessitating the appointment of another house surgeon. Funds are therefore urgently needed. A pleasing incident of the meeting was the notification that a grateful patient of the working classes had become an annual subscriber.

The St. Mary's Cottage Hospital at Southampton not only accomplishes the work of healing, but, according to the report, "encourages the patients to watch the dressing and bandaging, that they may take the knowledge to their distant homes." We also learn that "vicarious cures have been affected in other parts of the world by two simple papers of 'Plain Directions.'" (!)

The North Allerton Cottage Hospital has undergone a year of hard work, from the exceptional number of accidents obtaining admission. The funds are in a better condition, owing to the generosity of Mrs. Dixon. The new buildings provided by the Dundas Memorial Fund have been commenced, and the hospital premises will be leased to the committee for twenty-one years.

The Hospital Week Committee at Northampton have handed over £1,000 to the General Infirmary. By an unfortunate misunderstanding the Trades Unions did not co-operate the amount collected, therefore although appearing comparatively small, is very creditable. It is hoped that next year the co-operation of not only the Trades Unions but also of the whole county will greatly augment the collection.

The Grantham Hospital is benefited by a house-to-house collection annually. In connection with this organisation a dinner is held for the workers every year. On the last occasion of the festivity a collection of over £170 in favour of the hospital was announced. One worker present remarked that during the twenty years he had appealed for the hospital only twice had he been refused — an excellent testimony to the appreciation in which the hospital is held.

### The Student of Medicine.

### APPOINTMENTS.

J. S. Boden, L.R.C.P., M.R.C.S., appointed Assistant House Accoucheur to King's College Hospital. Dr. Bond, appointed Medical Officer of Health for Southwark. Surgeon-Major W. P. Carson, M.B., I.M.S., appointed Port Surgeon at Aden. Dr. C. Coles, appointed Medical Officer to the Leicester Dispensary. T. W. Astley Cooper, L.S.A., appointed House Surgeon to King's College Hospital. R. Crawfurd, M.A.Oxon., M.B., B.Ch., M.R.C.P., appointed Assistant House Physician to King's College Hospital. E. N. Fox, M.B.Vict., B.Ch., appointed Resident Medical Officer to the Manchester Hospital for Diseases of the Chest. L. P. Gamgee, F.R.C.S.Eng., appointed Surgeon to the Children's Hospital, Birmingham. J. Griffith, F.R.C.S., appointed Assistant Surgeon to the Royal Westminster Ophthalmic Hospital. L. W. A. Keiffenhem-Trubridge, M.B.Durh., appointed District Medical Officer and Medical Officer of the Workhouse of the Hoo Union. G. M. Macdonald, B.A.Cantab., M.R.C.S., L.R.C.P., appointed House Surgeon to King's College Hospital. E. J. Maclean, M.B., C.M.Edin., appointed Physician-Accoucheur to the St. Pancras and Northern Dispensary. G. S. Mill, M.B., C.M.Edin., appointed Honorary Surgeon to the Dewsbury Infirmary. Mr. E. F. Moon, appointed Assistant Medical Officer to the Derby Borough Lunatic Asylum. P. T. O'Sullivan, M.B., Ch., B.A.O.R.U.I., appointed Extern Surgeon to the South Charitable Infirmary and County Hospital, Cork. A. Philip, M.B., C.M.Edin., appointed Junior House Surgeon to the London Temperance Hospital, Hampstead Road, N.W. W. Ridley, M.B., M.S., F.R.C.S.Eng., appointed Assistant Surgeon to the Royal Infirmary, Newcastle-on-Tyne. F. C. Sprawson, M.R.C.S., L.R.C.P., appointed House Surgeon to King's College Hospital. F. S. Toogood, M.D.Lond., D.P.H., appointed Medical Superintendent of the New Infirmary at Lewisham. C. Wace, L.R.C.P., M.R.C.S., appointed House Physician to King's College Hospital. H. R. Wadd, M.R.C.S. Eng., L.R.C.P.Lond., appointed House Physician to the Great Northern Central Hospital. T. C. Webb, B.A.Cantab., M.R.C.S., L.R.C.P., appointed House Accoucheur to King's College Hospital. S. R. Wells, M.B., B.Sc.Lond., appointed Assistant Curator to St. George's Hospital.

### VACANCIES.

The following vacancies are announced this week, the amount of salary in each case being given after the name of the institution:

### Resident Medical Officers.

Birmingham and Midland Eye Hospital.—Assistant House Surgeon. Salary £50 per annum, with apartments and board. Applications to the Chairman of the Medical Board by December 13th.

Birmingham General Dispensary.—Resident Surgeon. Salary £150 per annum. Applications and testimonials to A. Forrest, Secretary, before December 12th.

City of London Hospital for Diseases of the Chest, Victoria Park, E.—House Physician. Appointment for six months. Board, residence, and allowance for washing provided. Applications to the Secretary at the Office, 24, Finsbury Circus, E.C., by December 13th.

Manchester Royal Infirmary.—Resident Medical Officer for the Fever Hospital at Monsall; doubly qualified, not less than 25 years of age. Salary £250 per annum, with board and residence. Applications to the Chairman of the Board by December 15th.

Royal Berks Hospital, Reading.—House Physician, House Surgeon, and Assistant Medical Officer. Salary £60 per annum each for the first two appointments, with board and lodging. For the third appointment board and lodging will be provided, but no salary. Applications to the Secretary before December 11th.

Sheffield General Dispensary.—Assistant House Surgeon; doubly qualified. Salary £80 per annum, with board, lodging, and washing. Appointment for three years. Applications to the Medical Staff of the Sheffield General Infirmary to the care of the Secretary by December 8th.

Warneford Hospital, Leamington Spa.—House Surgeon; single. Salary £100 per annum, with board, lodging, and washing. Applications and testimonials to J. Warren, Secretary, before December 5th.

THE STUDENT OF MEDICINE—*continued.*

Royal Hospital for Diseases of the Chest, City Road, E.C.—House Physician. Appointment for six months. Salary at the rate of £40 per annum, with board and lodging. Applications to the Secretary by December 5th.

West London Hospital, Hammersmith Road, W.—House Physician and House Surgeon; tenable for six months. Board and lodging are provided. Applications and testimonials to R. J. Gilbert, Secretary Superintendent, before December 12th.

**Non-Resident Medical Officers.**

Royal College of Surgeons of England.—Three Member of the Court of Examiners; must be F.R.C.S. Applications to the Secretary by December 5th.

West London Hospital, Hammersmith Road, W.—Assistant Anæsthetist (Honorary and Non-Resident). Applications and testimonials to R. J. Gilbert, Secretary Superintendent, before December 12th.

### HOSPITAL

An Institutional Journal of Medicine, Nursing & Philanthropy.

### On Saturday, Dec. 22nd,

WILL BE ISSUED A

### Special Double Christmas Number

Embellished with many attractive features, in addition to the usual contents, including

### *An extra 8pp. Supplement,*

AND A

### Handsome Presentation Plate

(BY SPECIAL PERMISSION)

OF

### H.R.H. The PRINCESS of WALES

An extra large edition will be printed, but as it is anticipated that the demand for this number will be very great, it is advisable to

**ORDER COPIES EARLY FROM YOUR NEWSAGENT.**

The Price will remain the same as usual:

### 2D.

On Sale at Messrs. W. H. SMITH & SONS' Bookstalls, and at all Newsagents throughout the Country.

*If you cannot obtain a copy, write direct to the Office—*

**428, STRAND, LONDON, W.C.**

